# Gut microbiota, dietary intakes and intestinal permeability reflected by serum zonulin in women

**DOI:** 10.1007/s00394-018-1784-0

**Published:** 2018-07-24

**Authors:** S. Mörkl, S. Lackner, A. Meinitzer, H. Mangge, M. Lehofer, B. Halwachs, G. Gorkiewicz, K. Kashofer, A. Painold, A. K. Holl, S. A. Bengesser, W. Müller, P. Holzer, S. J. Holasek

**Affiliations:** 1 Department of Psychiatry and Psychotherapeutic Medicine, Medical University of Graz, Graz, Austria; 2 Otto Loewi Research Center (for Vascular Biology, Immunology and Inflammation), Unit-Immunology and Pathophysiology, Medical University of Graz, Graz, Austria; 3 Institute of Pathology, Medical University of Graz, Graz, Austria; 4 Otto Loewi Research Center (for Vascular Biology, Immunology and Inflammation), Unit: Pharmacology, Medical University of Graz, Graz, Austria; 5 Clinical Institute of Medical and Chemical Laboratory Diagnostics, Medical University of Graz, Graz, Austria; 6 Gottfried Schatz Research Center (for Cell Signaling, Metabolism and Aging), Unit-Biophysics, Medical University of Graz, Graz, Austria; 7 Landeskrankenhaus Graz Südwest-Standort Süd, Graz, Austria

**Keywords:** Gut microbiota, Zonulin, Intestinal permeability, Diversity, *Ruminococcaceae*, *Faecalibacterium*, Dietary intakes

## Abstract

**Purpose:**

Increased gut permeability causes the trespass of antigens into the blood stream which leads to inflammation. Gut permeability reflected by serum zonulin and diversity of the gut microbiome were investigated in this cross-sectional study involving female study participants with different activity and BMI levels.

**Methods:**

102 women were included (BMI range 13.24–46.89 kg m^−2^): Anorexia nervosa patients (*n* = 17), athletes (*n* = 20), normal weight (*n* = 25), overweight (*n* = 21) and obese women (*n* = 19). DNA was extracted from stool samples and subjected to 16S rRNA gene analysis (V1–V2). Quantitative Insights Into Microbial Ecology (QIIME) was used to analyze data. Zonulin was measured with ELISA. Nutrient intake was assessed by repeated 24-h dietary recalls. We used the median of serum zonulin concentration to divide our participants into a “high-zonulin” (> 53.64 ng/ml) and “low-zonulin” (< 53.64 ng/ml) group.

**Results:**

The alpha-diversity (Shannon Index, Simpson Index, equitability) and beta-diversity (unweighted and weighted UniFrac distances) of the gut microbiome were not significantly different between the groups. Zonulin concentrations correlated significantly with total calorie-, protein-, carbohydrate-, sodium- and vitamin B12 intake. Linear discriminant analysis effect size (LEfSe) identified *Ruminococcaceae* (LDA = 4.163, *p* = 0.003) and *Faecalibacterium* (LDA = 4.151, *p* = 0.0002) as significantly more abundant in the low zonulin group.

**Conclusion:**

Butyrate-producing gut bacteria such as *Faecalibacteria* could decrease gut permeability and lower inflammation. The diversity of the gut microbiota in women does not seem to be correlated with the serum zonulin concentration. Further interventional studies are needed to investigate gut mucosal permeability and the gut microbiome in the context of dietary factors.

**Electronic supplementary material:**

The online version of this article (10.1007/s00394-018-1784-0) contains supplementary material, which is available to authorized users.

## Introduction

The permeability of the intestinal mucosa depends on the junctional complex between the intestinal enterocytes, which includes tight junction proteins that regulate the transport of ions and water between the gut lumen and the blood stream [1].

Increased gut permeability has been linked to diseases showing low-grade inflammation [2] caused by antigens trespassing the gut barrier which subsequently leads to an inflammatory immune response [3]. The serum protein zonulin, which was first described by Fasano et al., can be used as a peripheral marker to assess gut permeability [3, 4].

High serum concentrations of zonulin, indicating a leaky gut, have been identified in obesity [2] and patients with high fasting glucose [5]. Along with the composition of the gut microbiota, zonulin was shown to be related to dietary factors (such as Vitamin D) [6]. Low serum zonulin levels have further been observed in pregnant, overweight women showing high alpha-diversity (Chao-1 and number of observed species) [7]. However, there are no data available regarding non-pregnant women with different BMI values ranging from extremely lean to obese (including patients suffering from anorexia nervosa) and athletes.

There is emerging evidence that the gut microbiota plays an important role in regulating the permeability of the intestinal mucosa and that a change in the microbial community impacts on gut mucosal barrier function [8, 9]. Further, results from animal studies have shown that gut permeability can be affected by physical activity. For example, mice with activity-based anorexia showed higher intestinal permeability and histological changes of the intestinal mucosa [10]. Additionally, intense physical activity may damage the structure of the barrier and excessively upregulate permeability [11].

Against this background, we designed a cross-sectional study to investigate, for the first time, serum zonulin in women of different BMI groups and in athletes along with the gut microbiota diversity and composition.

The specific objectives of our study were: (1) to determine whether there are differences in the alpha-diversity and beta-diversity of the fecal microbiome in women with high and low zonulin, (2) to identify to what extent serum zonulin differs between different BMI groups and athletes and (3) to detect specific gut microbial genera related to high or low zonulin in women.

Given that there is a relationship between gut microbial composition, body weight, metabolic profile, and serum zonulin [2], we hypothesized that women with very high/low BMI and athletes would present with enhanced levels of zonulin. Furthermore, we hypothesized that women with high zonulin might show significantly lower levels of microbial alpha-diversity in comparison to women with low zonulin.

## Methods

### Participants

#### Recruitment and group characteristics

In total, 102 female participants were included in this cross-sectional study. All participants gave their written informed consent. This study was conducted according to the Helsinki Declaration and was part of the “energy sensing in anorexia nervosa” (ESAN) project which started in 2014. It was approved by the ethics committee of the Medical University of Graz (MUG-26-383ex13/14).

The study population comprised 17 patients with anorexia nervosa (AN), 25 normal weight (NW) women, 21 overweight (OW) women, 19 obese (OB) women and 20 female normal weight athletes (AT) (local competitive level handball-players).

AN patients were recruited from three psychiatric hospitals in Graz, Austria. Five of the included AN patients received high-energy nutritional supplements. All AN patients received standard pharmacotherapy. 20 AT were recruited from women sport teams. All other participants were recruited at the university campus of Graz.

Inclusion criteria were as follows: (1) all women aged between 18 and 40 years (2) AT with regular training schedule for at least 7 h per week (3) AN patients meeting the ICD-10 criteria in a structured diagnostic interview.

Exclusion criteria were: antibiotic or antifungal treatment within the previous 2 months, intake of prebiotics or probiotics within the previous 2 months (the consumption of dairy products and yogurt was permitted), regular intake of medication (except for AN patients), acute or chronic diseases or infections (including chronic inflammatory disorders, autoimmune disorders, acute fever) within the previous 2 months, drug or alcohol abuse, cognitive deficits, life-threatening conditions during AN, history of digestive diseases such as inflammatory bowel disease and irritable bowel syndrome, history of gastrointestinal surgery (other than appendectomy), pregnancy and period of breastfeeding.

### Assessment of body mass index (BMI)

Weight and height were measured with a calibrated digital stadiometer and platform-scale (Secca 764). BMI was calculated as: BMI = m/h^2^, in kg/m^2^. The groups “normal weight”, “overweight”, and “obese” were allocated according to the WHO BMI classification [12].

### Bioimpedance analysis (BIA)

Bioelectrical impedance analysis (BIA) is an easy-to-use, non-invasive and relatively inexpensive technique to estimate total body fat [13–15]. We used single frequency BIA (BIA 101—Body Impedance Analyzer Akern) as described in the manual [16], and analyzed the BIA output with the body composition software (BodyComposition—Professional v9.0.14325), using the equations from Sun [17] and the equations from Sergi [18].

### Ultrasound measurement of subcutaneous fat

An accurate ultrasound method, first described by Müller et al. (2016) was used to determine subcutaneous adipose tissue (SAT) at eight standardized measurement points [19]. This ultrasound method detects SAT without compression. The sites were marked relative to the individual’s body height. A semi-automatic image evaluation software (US Tissue-FAT, rotosport.at) was used to derive SAT at the measurement points and to calculate the sum of SAT thicknesses in mm (*D*_incl_). The index “incl” refers to thickness measurements which include the fibrous structures embedded in the SAT.

### Laboratory parameters

The blood draw was conducted in overnight-fasted participants. After blood sampling, plasma was centrifuged at 4000 r/min for 15 min and stored subsequently at − 80 °C for future determinations.

#### Zonulin

Serum zonulin was determined using a competitive ELISA kit (Immundiagnostik AG, Bensheim, Germany) according to the manufacturer’s instructions. The assay sensitivity was < 0.01  ng/ml. Intra- and interassay coefficients of variation were between 2.8 and 8.1% and between 4.8 and 11.6%. The ELISA kit detects the active (uncleaved) form of zonulin.

#### Serum lipids

Serum lipids (total cholesterol, triglycerides, LDL-cholesterol, HDL-cholesterol) were measured by enzymatic photometric methods (Roche Diagnostics, Mannheim, Germany). The limit of quantification (LOQ) for total cholesterol, HDL-cholesterol and triglycerides was 0.1 mmol/L. LDL-cholesterol concentrations were determined by Friedewald’s formula [20].

#### Markers of inflammation

C-reactive protein (CRP) and Interleukin (IL)-6 were analyzed with a particle-enhanced turbidimetric assay on a Cobas 6000 chemical routine analyzer (Roche Diagnostics, Mannheim, Germany). The limit of quantification (LOQ) for CRP was 0.25 mg/L. The intra-assay and inter-assay coefficients of variation (CV) of all routine assays were below 5%.

### Nutritional assessment

Two times repeated 24 h recalls [21] were performed by a qualified nutritionist. Dietary intake was quantified and analyzed by a nation-specific software and nutrition database (dato Denkwerkzeuge, Software: nut.s science, v1.32.44; Wien, 2010; http://www.nutritional-software.at).

### Microbiome analysis

The workflow for microbiome analysis with Ion torrent has already been described in detail in Mörkl et al. 2017 [22]. The PSP spin stool DNA stool collection kit (Stratec, Birkenfeld, Germany) was used for the collection of stool samples. One gram of the stool sample was suspended in the PSP-Spin-Stool-DNA-Plus-Kit-buffer-solution and immediately stored in a − 20 °C freezer. Sequence analysis was done according to the supplier’s recommendations. The PowerLyzer PowerSoil DNA Isolation Kit (MO BIO Laboratories Inc, CA, USA) was used to extract DNA according to manufacturer’s instructions. DNA concentration was measured by Picogreen-fluorescence (Thermo Fisher Scientific, MA, USA). The variable V1–V2 region of the bacterial 16S rRNA gene was amplified with polymerase chain reaction (PCR) from 50 ng fecal DNA using oligonucleotide primers *GATTGCCAGCAGCCGCGGTAA* and *GGACTACCAGGGTATCTAAT* [23]. 16S rRNA was amplified with the Mastermix 16S Complete PCR Kit (Molzym, Bremen, Germany). The first PCR reaction product was subjected to a second round of PCR with primers fusing the 16S primer sequence to the A and P adapters necessary for Ion Torrent sequencing including a molecular barcode sequence to allow multiplexing of up to 96 samples simultaneously. PCR products were subjected to agarose gel electrophoresis and the band of the expected length (about 330 nt) was excised from the gel and purified using the QiaQick gel extraction system (Qiagen, Hilden, Germany). Picogreen-fluorescence was used to measure the DNA concentration of the final PCR product.

Amplicons from up to 60 samples were pooled equimolarly and subjected to emulsion PCR using the Ion Torrent One Touch 2.0 Kit according to manufacturer’s protocols. After emulsion PCR, the beads were purified on Ion ES station and loaded onto Ion Torrent 318 chips for sequencing. Sequencing reactions were done on Ion Torrent PGM using the Ion 400BP Sequencing Kit running for 1082 flows (all reagents from Thermo Fisher Scientific, MA, USA). Sequences were split by barcode and transferred to the Torrent suite server for further analysis. Unmapped bam files were used as input for bioinformatics.

Initially, sequences were trimmed by a sliding window quality filter with a width of 20 nt and a cutoff of Q20. Reads shorter than 100 nucleotides and reads mapping to the human genome were removed with Deconseq [24]. Error correction was done using the Acacia tool [25] leading to error correction of 10–20% of reads. PCR chimeras were removed by the USearch algorithm. QIIME 1.8-workflow-scripts [26] for open-reference operational taxonomic unit (OTU) picking were used to analyze the final sequences. Based on a 97% similarity level, sequences were clustered into OTUs.

### Statistical analysis and visualization

The analyses were conducted in SPSS V23.0 (IBM, Paris, France) and R version 2.14.0 (R-foundation, Vienna, Austria). Data visualization was performed using GraphPad-Prism v5. Unless stated otherwise, descriptive results of continuous variables are expressed as mean and standard deviation (SD) for Gaussian-distributed variables.

The median of serum zonulin concentration was used to divide our sample into a “high-zonulin” (> 53.64 ng/ml) and “low-zonulin” (< 53.64 ng/ml) group. The median was calculated and the interquartile range (IQR) is given in brackets. In a second step, the participants were divided in three groups of 34 persons each dependent on their zonulin level (low third-zonulin group (0–44.55 ng/ml); medium third-zonulin group (45.06–62.29 ng/ml); high third-zonulin group (64.35–144.22 ng/ml)). To measure and compare levels of alpha-diversity between the high- and the low-zonulin group, number of observed species, Chao-1-diversity index, equitability and Shannon index were calculated with QIIME’s 1.9.1 alpha_diversity script using default settings [26] on the galaxy-server of the Medical University of Graz (galaxy.medunigraz.at). Depending on the distribution of data, we performed an ANOVA, Student’s t test or a Mann–Whitney *U* test to identify differences between groups. Levels of statistical significance were set at *p* < 0.05. Beta-diversity indices between samples were calculated based on weighted and unweighted UniFrac distance matrices [27]. Principal coordinate analysis (PCoA) was used to graphically identify different microbial community structures. The relation between variables was analyzed by Spearman’s correlation coefficient. Linear discriminant analysis Effect Size (LEfSe) [28] was used to identify differentially abundant taxa within the high- and the low-zonulin groups. The study data have been uploaded in the European Nucleotide Archive (ENA) under the study accession number PRJEB25022.

## Results

### Serum zonulin concentrations

The mean age of all the participants (*n* = 102) was 24.6 ± 4.6 (SD) years and mean BMI of all participants was 24.28 ± 6.5. The mean age of disease onset of AN-patients (six had the purging type of AN) was 21.79 ± 3.62 years. The mean duration of AN in patients was 3.14 ± 3.51 years. AN patients had a mean BMI of 15.22 ± 1.27, normal weight participants had a mean BMI of 21.94 ± 1.75, overweight participants had a mean BMI of 27.86 ± 1.08, adipose participants had a mean BMI of 34.66 ± 4.52 and athletes had a mean BMI of 22.14 ± 1.76.

Serum zonulin values ranged from 0 to 144.22 ng/ml in all participants (*n* = 102). The median (IQR) concentration of zonulin was 53.64 ng/ml (26.91).

**Fig. 1:**
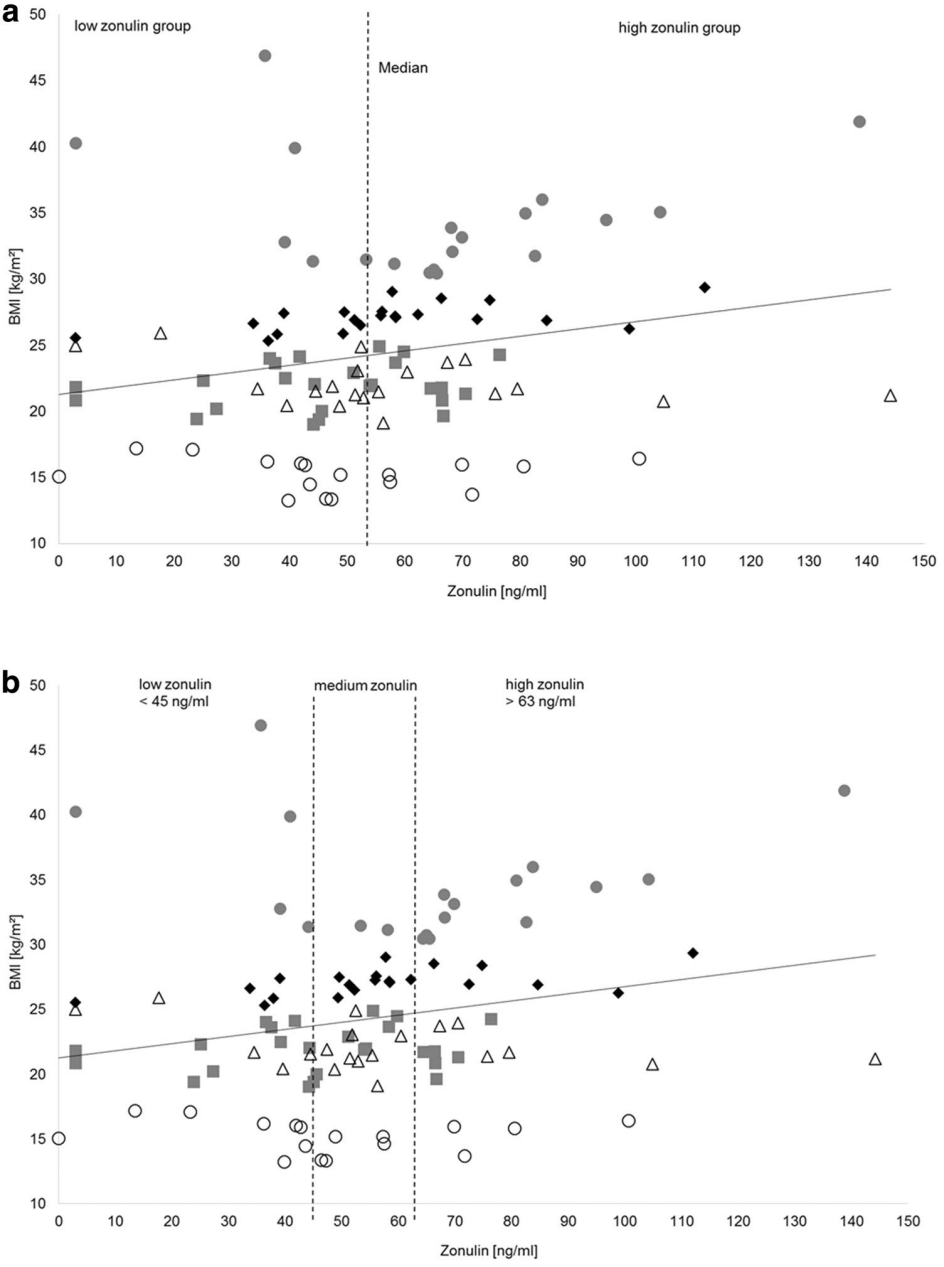
Zonulin distribution of study participants. The dashed line indicates the median of zonulin concentration (53.64 ng/ml) which divides the study participants into a low-zonulin group (< 53.64 ng/ml) and a high-zonulin group (> 53.64 ng/ml). Gray squares symbolize normal weight participants, black diamonds symbolize overweight participants, gray dots symbolize obese participants, white circles symbolize anorexia nervosa patients, black triangles symbolize athletes

We used the median of zonulin (53.64 ng/ml) to divide the recruited participants into a high-zonulin (> 53.64 ng/ml) and a low-zonulin (< 53.64 ng/ml) group. The median zonulin concentrations were 40.91 ng/ml (IQR 13.65) in the low- and 67.38 ng/ml (IQR 22.16) in the high-zonulin group. Six out of 17 (35.29%) AN patients, 13 out of 19 (68.42%) obese, 12 out of 21 (57.14%) overweight, 11 out of 25 (44.00%) normal weight participants and 9 out of 20 (45.00%) athletes belonged to the high-zonulin group (Fig. 1a).

The high- and low-zonulin groups showed no significant difference regarding age [*F*(19,82) = 1.52, *p* = 0.183] but a trend towards significance regarding BMI [*F*(1,100) = 3.66, *p* = 0.059] (Table 1). Further, there was a significant difference of waist circumference between the high- and low-zonulin group [*F*(1,100) = 5.85, *p* = 0.017]. There was also a trend towards significance regarding total fat mass measured with BIA [*F*(1,100) = 3.73, *p* = 0.056] between the high- and the low-zonulin group. Further, there was a significant difference of SAT(*D*_incl_) measured by ultrasound between the high- and the low-zonulin group [*F*(100,1) = 8.732, *p* = 0.004].

**Table 1:** Characteristics of the high- (> 53.64 ng/ml) and low-zonulin group (< 53.64 ng/ml) derived from division by the median (53.64 ng/ml) of serum zonulin concentration

Characteristics of the high- and low-zonulin group	Low-zonulin group (< 53.64 ng/ml) *n* = 51	High-zonulin group (> 53.64 ng/ml) *n* = 51	*p* value
Age (years)	23.98 (3.62)	25.19 (5.36)	0.183
Body mass index (kg m^−2^)	23.06 (6.78)	25.52 (6.21)	0.059
Waist hip ratio	0.74 (0.05)	0.76 (0.06)	0.024*
Waist circumference (cm)	72.57 (12.33)	78.50 (12.43)	0.017
Hip circumference (cm)	97.72 (15.47)	102.49 (13.79)	0.103
HDL-Cholesterol (mg/dl)	77.01 (18.35)	72.12 (15.16)	0.154
LDL-Cholesterol (mg/dl)	88.49 (29.59)	87.41 (30.31)	0.856
Triglycerides (mg/dl)	83.75 (43.97)	99.37 (46.94)	0.086
CRP (mg/l)	2.05 (2.67)	3.94 (4.77)	0.015*
IL-6 (pg/ml)	2.03 (0.92)	2.80 (1.60)	0.003*
Zonulin (ng/ml)	35.56 (15.44)	73.48 (20.05)	< 0.001*
Fat mass measured with bioimpedance analysis (%)	27.48 (12.09)	31.95 (11.27)	0.056
Sum of subcutaneous adipose tissue (SAT) (*D*_incl_) (mm)	81.32 (69.01)	120.11 (63.44)	0.004*
Number of observed species	653.20 (127.13)	630.71 (134.29)	0.387
Chao-1-diversity	1402.94 (355.77)	1339.03 (321.25)	0.343
Equitability	0.68 (0.04)	0.68 (0.05)	0.233
Shannon Index	6.42 (0.48)	6.29 (0.59)	0.240

Values are given as means and standard deviations (SD). Depending on the distribution of data, we performed an ANOVA or a *Kruskal-Wallis-Test* to identify differences between groups

*p < 0.05

ANOVA revealed significant differences of CRP (*p* = 0.015) and IL-6 (*p* = 0.003). Table 1 shows group characteristics of the high- and the low-zonulin group.

When the participants were segregated according to BMI [12], no significant group differences of zonulin levels could be detected [*F*(4,97) = 2.12, *p* = 0.084] among the AN patients (*n* = 17), NW participants (*n* = 25), OW participants (*n* = 21), OB participants (*n* = 19) and normal weight athletes (*n* = 20) (Table 1).

As many women were close to the median of zonulin (Fig. 1a), the group was in a second step divided in thirds to allow a clearer segregation between high- and low-zonulin levels (Fig. 1b). Therefore, we got three groups of 34 participants each (low-, medium- and high zonulin) (Table 2). Participants of the high-zonulin group were significantly older than participants of the medium-zonulin group (*p* = 0.041).

**Table 2:** Characteristics of the high-third-, the medium-third- and the low-zonulin group

Characteristics of the high-third-, the medium-third- and the low-third-zonulin group	Low-third zonulin (*n* = 34)	Medium-third zonulin (*n* = 34)	High-third zonulin (*n* = 34)	*p* value
Age (years)	24.38 (3.79)	23.32 (3.18)	26.06 (6.01)	0.045*
Body mass index (kg m^2^)	23.57 (7.54)	22.98 (4.81)	26.32 (6.81)	0.082
Waist hip ratio	0.75 (0.44)	0.74 (0.48)	0.77 (0.06)	0.118
Waist circumference (cm)	73.48 (14.14)	72.92 (8.91)	80.21 (14.35)	0.029*
Hip circumference (cm)	97.74 (16.61)	98.34 (11.63)	104.32 (15.19)	0.135
HDL-cholesterol (mg/dl)	74.47 (17.71)	80.59 (17.48)	68.65 (13.58)	0.013*
LDL-cholesterol (mg/dl)	85.23 (24.78)	86.74 (32.09)	91.88 (32.41)	0.633
Triglycerides (mg/dl)	82.71 (45.74)	86.41 (39.65)	105.59 (49.81)	0.087
CRP (mg/l)	2.38 (3.08)	2.25 (3.54)	4.36 (4.82)	0.048*
IL-6 (pg/ml)	2.13 (1.01)	1.97 (0.84)	3.15 (1.74)	< 0.001*
Zonulin (ng/ml)	29.99 (14.98)	53.57 (4.59)	81.52 (20.18)	< 0.001*
Fat mass measured with bioimpedance analysis (%)	28.05 (12.16)	28.79 (11.66)	32.30 (11.61)	0.290
Sum of subcutaneous adipose tissue (SAT) (*D*_incl_) (mm)	85.33 (74.10)	88.96 (55.74)	127.87 (68.79)	0.017*
Chao-1-diversity	1405.61 (342.03)	1447.71 (350.79)	1259.63 (301.96)	0.054
Equitability	0.69 (0.03)	0.69 (0.04)	0.67 (0.05)	0.054
Shannon Index	6.40 (0.44)	6.51 (0.53)	6.18 (0.60)	0.066

Values are given as means and standard deviations (SD). Depending on the distribution of data, we performed an ANOVA or a test to identify differences between groups

*p < 0.05

Four out of 17 AN patients, 12 out of 19 obese, 6 out of 21 overweight, 6 out of 25 normal weight participants and 6 out of 20 athletes belonged to the high-zonulin group (Fig. 1b).

The high- and the low-third-zonulin groups showed no difference regarding age [*t*(66) = − 1.375, *p* = 0.175], BMI [*t*(66) = − 1.58, *p* = 0.119] and total fat mass measured with BIA [*t*(66) = − 1.393, *p* = 0.168]. However, there was a significant difference of SAT (*D*_incl_) measured by ultrasound between the high third and the low-third-zonulin group [*t*(66) = − 2.45, *p* = 0.017]. Further, there was a significant difference of waist circumference [*t*(66) = − 2.02, *p* = 0.048]. Additionally, we found a significant correlation between serum zonulin and waist circumference (*r* = 0.263, *p* = 0.007).

The high-third zonulin group showed significantly more CRP compared to the low-third zonulin group [*t*(66) = − 2.011, *p* = 0.048] and significantly more IL-6 [*t*(66) = − 2.939, *p* = 0.005].

Table 2 depicts the group characteristics of the high-third, the medium-third and the low-third-zonulin group.

Further, there were no significant differences of serum zonulin between athletes (*n* = 20) and non-athletes (AN patients, NW participants, OW participants, OB participants; *n* = 82) [*t*(100) = − 0.56, *p* = 0.573].

### Correlations between dietary components  and serum zonulin

We detected differences in dietary intakes between the participants with high and low-serum zonulin (Table 3). These differences were related to significantly higher absolute intakes of total calories, total protein, total carbohydrates and total fat.

**Table 3:** Dietary components of the low and the high zonulin group

Dietary components	Low-zonulin group (< 53.64 ng/ml) *n* = 51	High-zonulin group (> 53.64 ng/ml) *n* = 51	*p* value
Total food amount (g)	4349.11 (4313.35)	4195.79 (4423.47)	0.860
Total calorie intake (kcal)	1779.01 (624.71)	2068.42 (603.35)	0.019*
Total protein intake (g)	64.57 (24.53)	75.43 (25.94)	0.032*
Total carbohydrate intake (g)	201.99 (84.34)	237.95 (88.51)	0.038*
Total fibre intake (g)	22.12 (12.32)	20.40 (7.33)	0.394
Total fat intake (g)	74.25 (79.90)	85.10 (27.18)	0.032*
Vitamin B12 intake (µg)	3.19 (1.58)	4.14 (2.13)	0.017*
Sodium intake (mg)	2402.98 (974.17)	2933.18 (1302.45)	0.022*

The amount of dietary components is calculated from the mean of the two recorded days and represents the average consumed amount per day. Values are given as means and standard deviations (SD). Depending on the distribution of data, we performed an ANOVA or a Mann–Whitney *U* test to identify differences between groups

*p < 0.05

Spearman’s correlations showed small correlations  between zonulin and total calorie intake (*r* = 0.230, *p* = 0.036), protein intake (*r* = 0.208, *p* = 0.036), carbohydrate intake (*r* = 0.221, *p* = 0.025), sodium intake (*r* = 0.207, *p* = 0.037) and vitamin B12 intake (*r* = 0.198, *p* = 0.046). There was no significant correlation between zonulin and fat intake (*r* = 0.183, *p* = 0.065). Additionally, we compared dietary intakes of the high (*n* = 34) and low-third (*n* = 34)-zonulin group (Table 4). Interestingly, no significant differences could be detected.

**Table 4:** Dietary components of the high third-, the medium third- and the low-third-zonulin group

Dietary components	Low-third zonulin (*n* = 34)	Medium-third zonulin (*n* = 34)	High-third zonulin (*n* = 34)	*p* value
Total food amount (g)	4530.05 (5219.72)	4666.19 (5381.22)	3621.11 (993.27)	0.564
Total calorie intake (kcal)	1761.88 (700.25)	1939.55 (598.32)	2069.72 (556.82)	0.128
Total protein intake (g)	62.45 (25.93)	70.40 (24.46)	77.16 (25.32)	0.060
Total carbohydrate intake (g)	207.57 (95.53)	217.94 (93.74)	234.42 (73.18)	0.451
Total fibre intake (g)	20.42 (10.43)	23.29 (12.42)	20.06 (6.79)	0.357
Total fat intake (g)	71.02 (32.43)	82.13 (23.06)	85.88 (29.30)	0.088
Vitamin B12 intake (µg)	3.08 (1.71)	3.82 (1.96)	4.09 (2.29)	0.104
Sodium intake (mg)	2560.69 (1139.32)	2641.90 (1375.96)	2801.65 (1001.53)	0.690

The amount of dietary components is calculated from the mean of the two recorded days and represents the average consumed amount per day. Values are given as means and standard deviations (SD). Depending on the distribution of data, we performed an ANOVA or a Mann–Whitney *U* test to identify differences between the low-third and the high-third zonulin group

*p < 0.05

### Association of gut microbiota with serum zonulin

A total of 4.988.322 sequences with an average of 48.905 (range 14,756–120,406) were obtained after quality filtering and removal of chimeric reads.

No significant differences in measures of alpha-diversity such as number of observed species [*F*(1,100) = 0.75, *p* = 0.387], Chao-1-diversity index [*F*(1,100) = 0.91, *p* = 0.343], equitability [F(1,100) = 1.44, *p* = 0.233] and Shannon index [F(1,100) = 1.40, *p* = 0.240] were detected between the high and low-zonulin group (Table 1). There were also no significant differences between the high-third- and the low-third-zonulin group (Table 2).

Overall, there was no significant difference on phylum level of gut microbiota between the high and low-zonulin group. The results of overall composition of gut microbiota with relative abundances of phyla are depicted in Supplementary Fig. 1 and Supplementary Fig. 2.

**Fig. 2:**
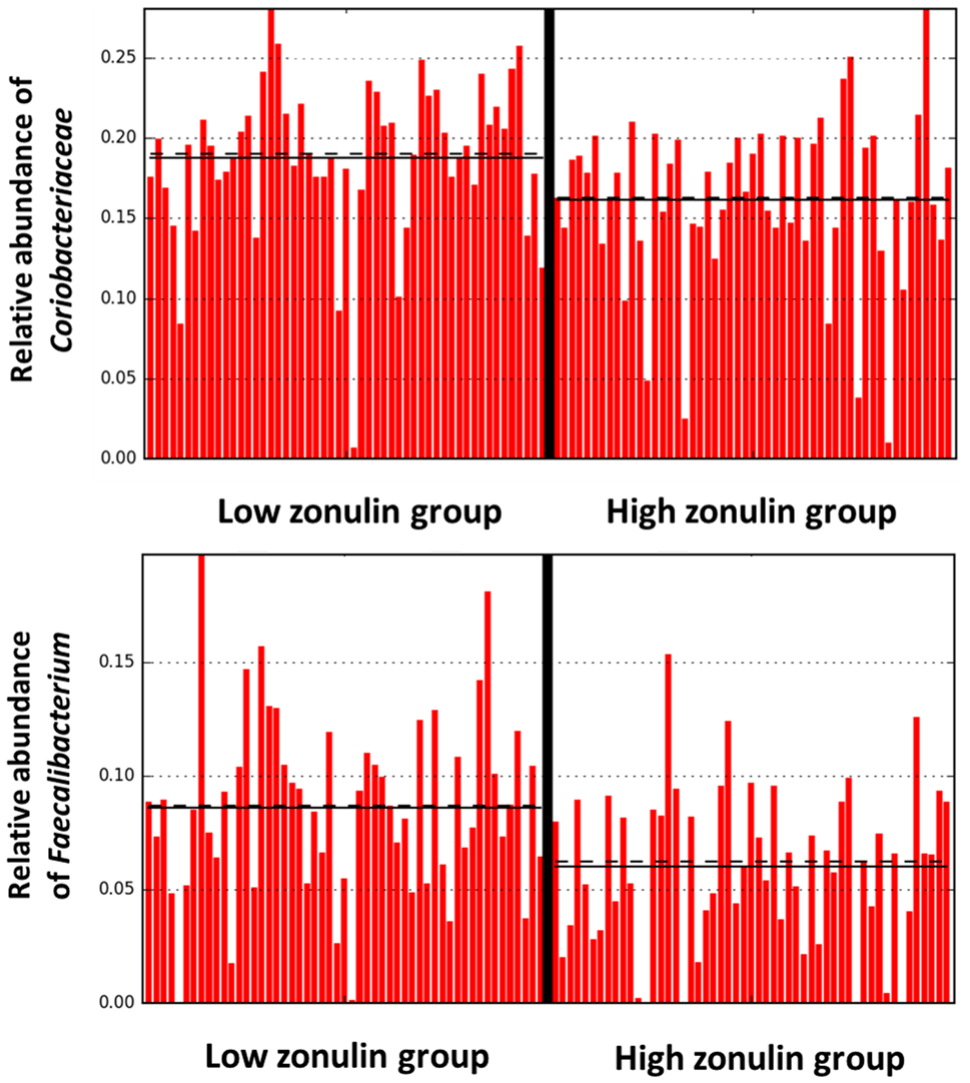
Relative abundances of *Ruminococcaceae* and *Faecalibacterium* in the high- (> 53.64 ng/ml) and low (< 53.64 ng/ml)-zonulin group

On family and species level, LEfSe analysis identified *Ruminococcaceae* (LDA = 4.163, *p* = 0.003) and *Faecalibacterium* (LDA = 4.151, *p* = 0.0002) as significantly more abundant in the low-zonulin group. Figure 2 shows the abundances of *Ruminococcaceae* and *Faecalibacterium* in the high- and low-zonulin group.

Additionally, when LEfSe analysis was performed between the high-third- and the low-third zonulin group, the following discriminative features were detected (total abundances in percental range): *Odoribacter* (LDA = 3.982, *p* = 0.049) and *Rikenellaceae* (LDA = 4.422, *p* = 0.036) were significantly higher in the low-third-zonulin group, whereas the class of *Erysipelotrichi* (LDA = 4.670, *p* = 0.034) and the order of *Erysipelotrichales* (LDA = 4.670, *p* = 0.034) were significantly higher in the high-third-zonulin group.

In a second step, using the median, we divided all women into a high vitamin B12 and low vitamin B12 group. *Archea* (LDA = 3.836, *p* = 0.038), *Odoribacteriaceae* (LDA = 3.448, *p* = 0.031), *Clostridia* (LDA = 4.236, *p* = 0.003) and *Proteobacteria* (LDA = 4.010, *p* = 0.042) were identified as differentially abundant features.

Further, LeFse analysis identified *Ruminococcaceae* (LDA = 4.280, *p* < 0.001) as a differentially abundant feature between the high and the low vitamin B12 group. Therefore, *Ruminococcaceae* were more abundant in the low-zonulin group and low vitamin B12 group.

Beta-diversity indices between samples were calculated based on weighted and unweighted UniFrac distance matrices [27] between the high and the low-zonulin group and the high-third- and the low-third-zonulin group. PcoA plots were used to visualize differences of community structures (Supplementary Fig. 3 and Supplementary Fig. 4). No significantly different community structures could be identified between the high and the low-zonulin group with unweighted (*p* = 0.965) and weighted (*p* = 0.208) UniFrac distance. Additionally, there were no significant differences of community structures with unweighted (*p* = 0.909) and weighted (*p* = 0.448) UniFrac between the high-third- and the low-third-zonulin group.

### Spearman’s correlations  between anthropometric and laboratory parameters

The zonulin serum concentration showed positive correlations with BMI (*r* = 0.235, *p* = 0.017), total fat mass (%) measured with BIA (*r* = 0.205, *p* = 0.039), *D*_incl_ (*r* = 0.244, *p* = 0.013), waist circumference (*r* = 0.263, *p* = 0.007), hip circumference (*r*= 0.231, *p* = 0.202), CRP (*r* = 0.293, *p* = 0.003), IL-6 (*r* = 0.317, *p* = 0.001) and triglycerides (*r* = 0.283, *p* = 0.004). The group readouts are listed in Tables 1 and 2.

Correlations between total abundances of *Ruminococcaceae* and *Faecalibacterium * and  CRP and IL-6 levels were calculated using Spearman’s correlations. However, no significant correlations between the investigated parameters could be identified.

## Discussion

To our knowledge, this is the second study to measure serum zonulin and gut microbiota diversity in women and the first one to include non-pregnant women from different BMI groups and athletes. We found significant correlations between serum zonulin and BMI, waist and hip circumferences and fat mass (measured by BIA and ultrasound). Further, the high-zonulin group showed significantly increased inflammation markers such as CRP and IL-6. Interestingly, there were no significant differences of species diversity of the gut microbiome and gut microbiota-community structures between the high- and low-zonulin groups. There were significant differences of dietary intakes between the high- and low-zonulin group, which were not confirmed when we compared the high-third- and the low-third-zonulin group.

Zonulin has been reported to be enhanced in overweight pregnant women [7] where significant differences in microbial alpha-diversity could be shown between a high and a low-zonulin group. Since microbial alpha-diversity did not significantly differ between the high and low-zonulin groups investigated in this study, bacterial diversity cannot be solely responsible for strengthening the gut barrier. In contrast, alpha-diversity was shown to be significantly different between BMI groups and athletes [22]. Nevertheless, there were no significant differences regarding serum zonulin between BMI groups and athletes in the current study.

While only 35.29% of AN patients belonged to the high-zonulin group, 68.42% of obese participants and 57.14% of overweight participants showed high-zonulin levels. It is noteworthy, that BMI alone may not account for high-zonulin levels indicating low gut barrier integrity. This was also underlined by the fact that the high- and low-zonulin groups were not different with regard to BMI and BMI showed only a modest correlation with serum zonulin levels (*r* = 0.235, *p* = 0.017). As BMI is not a sensitive measure of obesity, it is of note that we found significant differences of waist circumference between the high and low-zonulin group (*p* = 0.017) and the high-third- and the low-third-zonulin group (*p* = 0.048). Adding to this, there was a significant correlation between waist circumference and serum zonulin (*r* = 0.263, *p* = 0.007) confirming the results of a former study by Ohlsson which has shown impaired gut barrier in obese patients and a significant correlation between zonulin and waist circumference [29].

The interplay between gut microbiota and intestinal permeability might be crucial for proinflammatory responses, subsequently leading to a range of metabolic diseases [30]. For example, in our cohort, participants with high zonulin showed significantly higher values of CRP and IL-6. Consequently, it may be of importance which bacteria are present and what amounts of anti-inflammatory metabolites, such as short chain fatty acids (SCFAs) [31] they are capable of producing.

For example, *Ruminococcaceae* were significantly more abundant in the low-zonulin group, which indicates that they may contribute to intestinal barrier integrity. Interestingly, *Ruminococcaceae* belong to a group of common gut microbes which break down complex carbohydrates and are typically more abundant in people with diets high in carbohydrates [32, 33]. In our study, however, the high-zonulin group consumed significantly more carbohydrates, while *Ruminococcaceae* were less abundant in the high-zonulin group.

Given that low zonulin reflects low gut permeability, our findings indicate that *Ruminococcaceae* have a protective effect on gut barrier integrity. This is underlined by the results of a murine study in which the intake of carbohydrates led to an increase of *Ruminococcaceae* along with modulation of gut barrier function [34]. Nevertheless, when the group was divided in thirds with 34 participants each, there was no significant difference of *Ruminococcaceae* between the high-third (*n* = 34) and low-third (*n* = 34)-zonulin group, which may be because of group size.

*Faecalibacterium* was also more abundant in the low-zonulin group. *Faecalibacteria* are gram-negative SCFA producing *Firmicutes*, which have anti-inflammatory properties [35]. *Faecalibacteria* also display remarkably low abundances in diseases affecting internal and external barrier function, for example Crohn’s disease [36] and atopic dermatitis [37]. Therefore, low counts of *Faecalibacteria* could weaken the gut lining leading to inflammatory responses. *Odoribacter* and *Rikenallaceae* were predominantly abundant in the low-third-zonulin group. *Odoribacter* are also known for their SCFA (butyrate-) production [38]. An increase in taxa within *Faecalibacterium, Ruminococcaceae* and *Rikenellaceae* has been described in colonization resistance against *Clostridium difficile* in patients [39]. In general, those butyrate-producing bacteria are as well reduced in inflammatory bowel disease compared to healthy individuals [40–42]. This suggests that butyrate has a strengthening effect on the gut barrier, which was also demonstrated in experimental studies [43, 44] and highlights the important role of butyrate to prevent inflammation [45]. Therefore, the total amount of butyrate produced by gut bacteria seems to be of more importance than the abundance of bacterial species, families and phyla [46, 47].

As the composition of the gut microbiome is closely related to dietary components, its linkage to zonulin levels is of high interest. To our knowledge, no clinical study has yet investigated the impact of diet on intestinal permeability in different BMI groups including AN patients and a group of athletes. The high-zonulin group consumed significantly more calories, protein and fat, while the groups did not differ in total grams of food eaten but differed in total caloric intake. Mokkala et al., who investigated serum zonulin concentrations in overweight women in early pregnancy, detected a negative correlation between serum zonulin and protein intake [7] and suggested that proteins may have a positive impact on intestinal barrier function. On the contrary, the high-zonulin group in our study reported a significantly higher protein and vitamin B12 intake compared to the low-zonulin group. Dietary protein components are known to cause significant changes in metabolites of gut bacteria such as SCFAs, ammonia, hydrogen, sulfide and methane. These metabolites may act as cytotoxic agents and have been associated with inflammatory bowel diseases and colon cancer [48]. Hence, low-protein diets are often recommended to these patients, because a diet high in protein and low in carbohydrates has been shown to increase the risk of diseases by inducing the growth of pathogens and protein-fermenting bacteria. Summarized, by affecting the gut barrier, a diet-induced dysbiosis may, therefore, stimulate immune-mediated inflammation [48]. In an interventional study with a Nordic diet zonulin levels in serum tended to correlate positively with energy percentage of protein and inversely with energy percentage of carbohydrates [29]. They concluded, that higher protein content in food may trigger inflammation.

Our study results confirm the results of Zak-Golab et al. (2013), which show a correlation between serum zonulin and total calorie intake associated with higher fat intake in the high-zonulin group [2]. In our study, serum zonulin correlated with carbohydrate intake, however, there was no correlation between zonulin and fibre intake. Further, the total fibre intake did not differ significantly between the high and low-zonulin group. It is of note that the general fibre consumption in Austria is low and the recommended daily amount of 30 g fibre per day is not commonly reached [49]. Due to this, fibre intake might not have reached significance between the high and the low-zonulin group. Nevertheless, epidemiological studies have shown that a diet high in fibre is associated with lower inflammation [50]. Therefore, more interventional studies are needed to investigate zonulin and fibre intake.

Adding to that, despite the significant difference of fat intake between the high and low-zonulin group, the difference of fat intake of the high-third and the low-third-zonulin group as well as the Spearman’s correlation between total fat intake and serum zonulin did not reach significance level. Human and animal studies demonstrated that bacterial metabolites, called lipopolysaccharides (LPS) cause inflammation through diffusing in the circulatory system in response to high-fat dietary intake [51]. Besides total fat intake, the quality and the structure of fat might as well affect gut permeability. The type and quality of fat was not assessed in this study.

Micronutrients might as well be of special importance for gut barrier integrity, given that a positive correlation between sodium and vitamin B12 intake and serum zonulin was found. This could be due to the presence of vitamin B12 in protein-rich food such as eggs, dairy products, meat and fish. Among others, LeFse analysis identified *Ruminococcaceae* as a differentially abundant feature between the high and the low vitamin B12 group. Vitamin B12 was significantly higher in the high-zonulin group. *Ruminococcaceae* were more abundant in the low-zonulin group and low vitamin B12 group. As for the chain of events, it might be reasonable, that vitamin B12 intake might increase zonulin levels through changing total abundances of *Ruminococcaceae*. However, as this was a cross-sectional study, longitudinal dietary intervention studies are needed to shed light on this hypothesized chain of events.

Another major influence factor which should be carefully considered in future studies is the effect of food combinations, which could lessen the unfavorable effects of dietary fat and protein such as flavonoids, prebiotics and probiotics. Furthermore, besides micro- and macronutrients, food additives such as emulsifiers should be carefully monitored in future studies as they could negatively influence the gut barrier [52].

A limitation of our study is that the participants remained on their usual, non-standardized diet. Another limitation is related to the method used to estimate dietary intake. The results of the dietary recalls could have been affected by over- or underreporting. Especially AN patients are known to overestimate their dietary intakes [53] while obese participants could have underestimated their caloric intake [54]. Although most of the study participants were medication free, all included AN patients remained on their usual pharmacotherapy (mainly selective serotonin reuptake inhibitors), which could have affected intestinal microbiota through their antimicrobial properties [55].

As stool samples were collected across the menstrual cycle of the participants, microbial alpha-diversity could have been affected by the modulatory influence of estrogen [56, 57], which is reabsorbed dependent on gut microbiota function [58]. Referring to this, Flores et al. (2012) have shown that estrogen levels correlate with microbiome diversity [59]. Hence, the individual’s menstrual cycle and the estrogen levels may have had an influence on gut microbiota and subsequently on zonulin levels. Third, we only included women in our study. To our best knowledge, there are no differences of serum zonulin between men and women [2, 60]. However, more and larger studies are needed to investigate whether our study results are transferable to men.

## Conclusions

Dietary components seem to affect intestinal barrier integrity as reflected by serum zonulin levels through complex interactions with the gut microbiota. While in our study the diversity of the gut microbiota was not associated with serum zonulin, distinct butyrate-producing bacterial genera could act as anti-inflammatory mediators and regulate gut permeability.

Our findings provide a basis for future interventional studies to investigate serum zonulin as a biomarker of increased gut permeability in the context of nutrition. As diet enhancement and a modification of the gut microbiota could strengthen the intestinal barrier and damp immune activation, our results are of special importance for the metabolic health of women.

## Electronic supplementary material

Below is the link to the electronic supplementary material.

**Supplementary Fig.1**: Results of overall composition of gut microbiota with relative abundances [%] of phyla of the high (1) and the low (2) zonulin group (TIFF 619 KB)

**Supplementary Fig. 2**: Results of overall composition of gut microbiota with relative abundances [%] of phyla of the high (1) and the medium (2) and the low (3) zonulin group (TIFF 1335 KB)

**Supplementary Fig. 3 A/B**: Principal component analysis (PCoA) of the high (red) and low (blue) zonulin group. Each dot symbolizes the bacterial community composition of one individual stool sample. Axis titles indicate the percentage of the explained variation. Part A (left) shows the data for unweighted UniFrac distances, part B (right) for weighted UniFrac distances (TIFF 250 KB)

**Supplementary Fig. 4 A/B**: Principal component analysis (PCoA) of the high (red), medium (blue) and low (green) zonulin group. Each dot symbolizes the bacterial community composition of one individual stool sample. Axis titles indicate the percentage of the explained variation. Part A (left) shows the data for unweighted UniFrac distances, part B (right) for weighted UniFrac distances (TIFF 259 KB)
