# Supplementary figures and images for: Gut microbiota, dietary intakes and intestinal permeability reflected by serum zonulin in women
Source: Eur J Nutr. 2018 Jul 24;57(8):2985–97. doi: 10.1007/s00394-018-1784-0 (PMC6267414; doi:10.1007/s00394-018-1784-0)

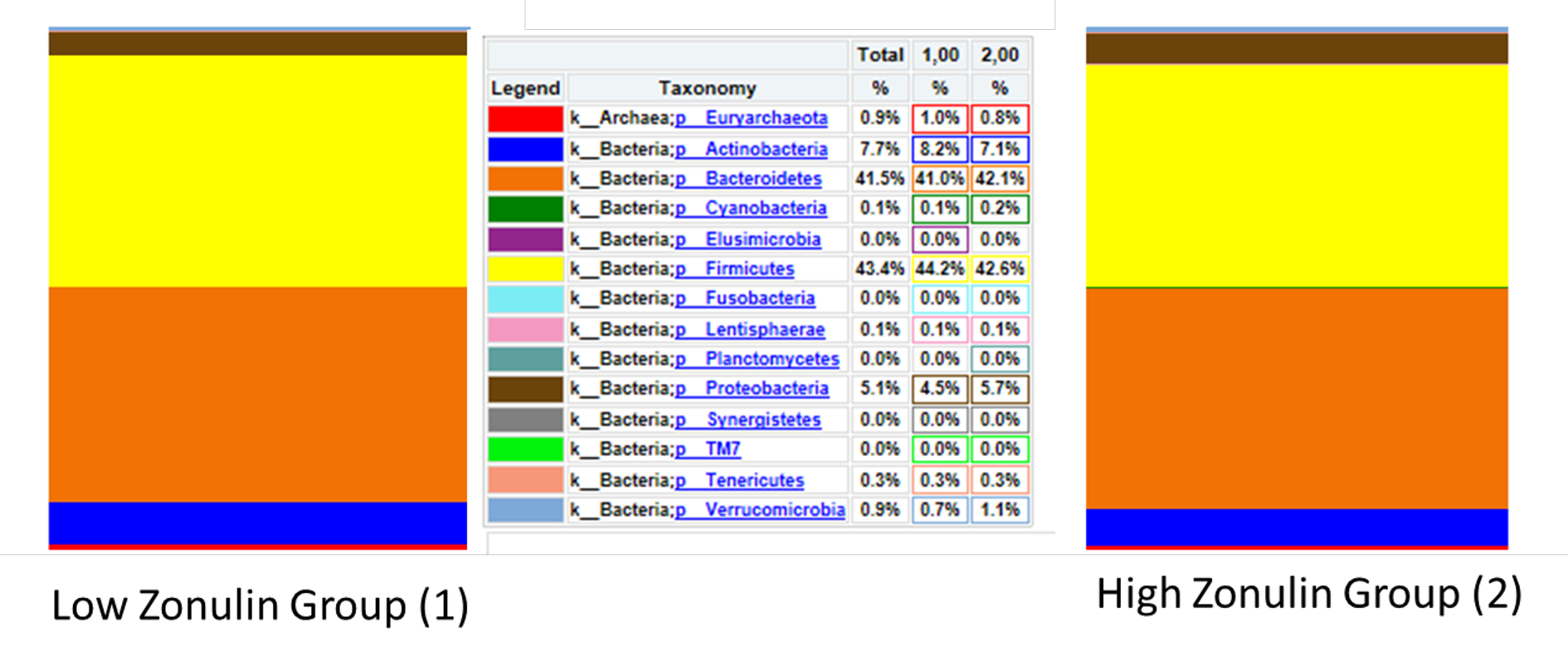

Supplement: Supplementary file 1 — Supplementary Fig.1: Results of overall composition of gut microbiota with relative abundances [%] of phyla of the high (1) and the low (2) zonulin group (TIFF 619 KB) [file 394_2018_1784_MOESM1_ESM.tiff]

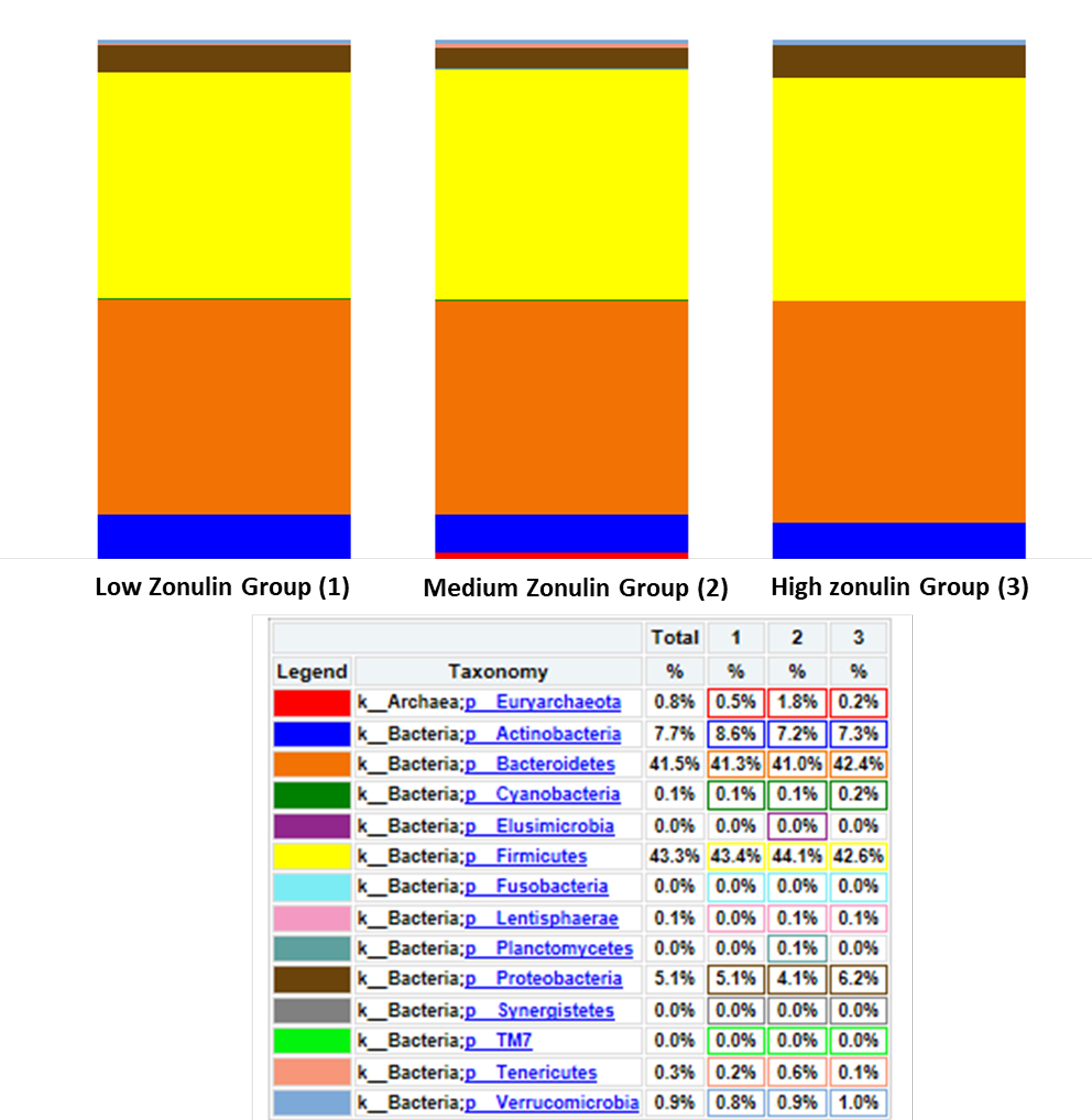

Supplement: Supplementary file 2 — Supplementary Fig. 2: Results of overall composition of gut microbiota with relative abundances [%] of phyla of the high (1) and the medium (2) and the low (3) zonulin group (TIFF 1335 KB) [file 394_2018_1784_MOESM2_ESM.tiff]

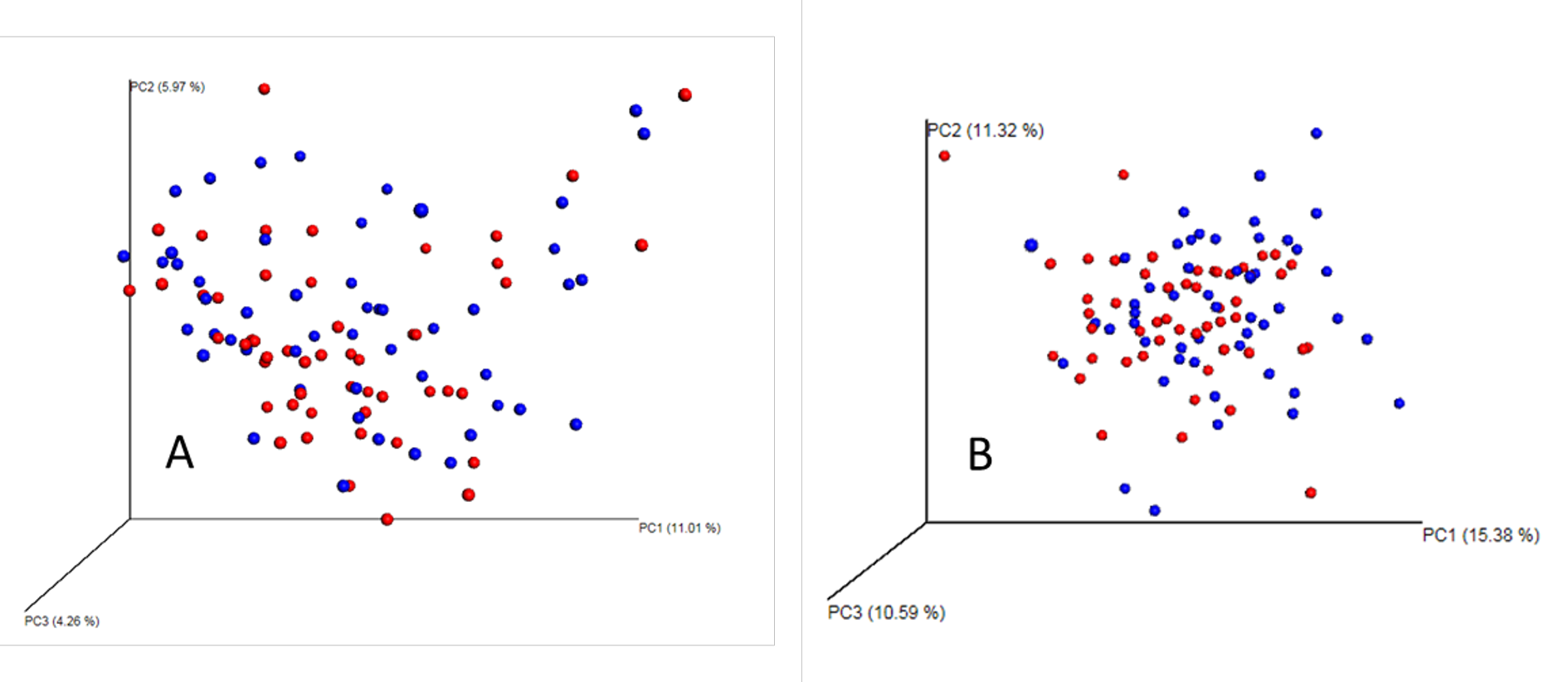

Supplement: Supplementary file 3 — Supplementary Fig. 3 A/B: Principal component analysis (PCoA) of the high (red) and low (blue) zonulin group. Each dot symbolizes the bacterial community composition of one individual stool sample. Axis titles indicate the percentage of the explained variation. Part A (left) shows the data for unweighted UniFrac distances, part B (right) for weighted UniFrac distances (TIFF 250 KB) [file 394_2018_1784_MOESM3_ESM.tiff]

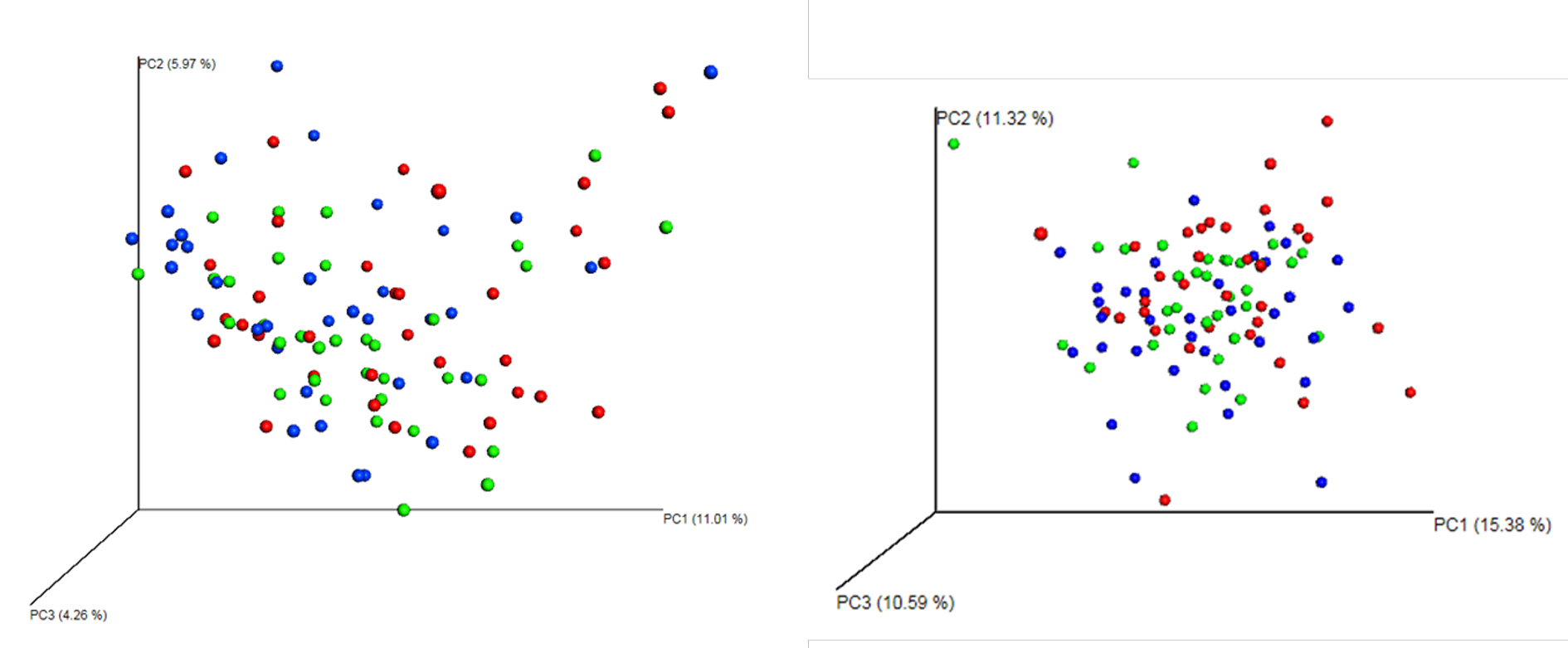

Supplement: Supplementary file 4 — Supplementary Fig. 4 A/B: Principal component analysis (PCoA) of the high (red), medium (blue) and low (green) zonulin group. Each dot symbolizes the bacterial community composition of one individual stool sample. Axis titles indicate the percentage of the explained variation. Part A (left) shows the data for unweighted UniFrac distances, part B (right) for weighted UniFrac distances (TIFF 259 KB) [file 394_2018_1784_MOESM4_ESM.tiff]
